# Efficacy Analysis of Separation Surgery Combined with SBRT for Spinal Metastases—A Long‐Term Follow‐Up Study Based on Patients with Spinal Metastatic Tumor in a Single‐Center

**DOI:** 10.1111/os.12594

**Published:** 2020-02-08

**Authors:** Liu Xiaozhou, Zhou Xing, Shi Xin, Li Chengjun, Zhang Lei, Zhou Guangxin, Wu Sujia

**Affiliations:** ^1^ Department of Orthopedics Jinling Hospital Nanjing China

**Keywords:** Efficacy analysis, SBRT, Separation surgery, Spinal metastatic tumor, Long‐term follow‐up study

## Abstract

**Objective:**

Follow‐up data of patients with spinal metastatic tumors were analyzed to investigate the effect of separation surgery combined with SBRT on clinical outcomes.

**Methods:**

The clinical data of 52 patients with spinal metastatic tumors admitted to our hospital from January 2015 to December 2018 were retrospectively analyzed. There were 24 males and 28 females, aged 25–77 years, with an average of 56.7 ± 7.4 years. The separation surgery of all patients was successfully completed and followed up. Frankel neurological function grading, Karnofsky performance scores, VAS scores, Epidural spinal cord compression (ESCC) grading and muscle strength grading were used to assess the patients’ condition. Kaplan‐Meier analysis and the Log⁃rank test were used to calculate the hazard ratio (*HR*) and the 95% feasible interval for patients with different ages, genders, and treatments. The multivariate Cox regression model was used to calculate the risk value HR and the 95% feasible interval in patients undergoing only separation surgery or separation surgery combined with SBRT.

**Results:**

After separation surgery, 46 patients had pain relief (88.5%), and the average VAS score decreased to 2.17 ± 0.52 points, which was significantly improved compared with preoperative score (*P* < 0.01). Muscle strength grading decreased in seven cases, showed no change in two cases, and recovered in 19 cases. Postoperative Frankel neurological function grading and Karnofsky performance scores were also significantly improved compared with preoperative scores (*P* < 0.01). The patients who accepted separation surgery were followed up for 9–47 months (26.3 ± 18.1 months), and 15 patients died due to the deterioration of the primary tumor. Thirteen patients received SBRT after surgery, including 12 cases of pain relief. The average VAS score of these 13 patients decreased to 1.64±0.41 points, which was significantly improved compared with preoperative and postoperative (*P* < 0.01), and muscle strength recovered in eight cases. Frankel neurological function grading and Karnofsky performance scores of these patients were also significantly improved compared with preoperative and postoperative Frankel neurological function grading and Karnofsky performance scores (*P* < 0.01). The patients who accepted separation surgery combined with SBRT were followed up for 11–38 months (mean 22.5 ± 10.2 months), and five cases died of primary tumor. Univariate and multivariate analysis showed that separation surgery combined with SBRT was an independent predictor of overall survival rate (OS).

**Conclusions:**

Separation surgery combined with SBRT is an effective way to treat spinal metastatic tumors as it not only has smaller surgical trauma, but can also significantly relieve pain, improve nerve function, and relieve spinal cord compression.

## Introduction

With the advancement of cancer diagnosis and treatment technology, and the use of targeted drugs, it is widely believed that the number of patients with malignant tumors in the terminal stage is significantly increased, and their survival is also significantly prolonged. In all patients with malignant tumors, spinal metastases with epidural spinal cord compression (ESCC) account for 40%^1^, accompanied by pain, pathological fractures, nerve root compression, and other symptoms. Therefore, the surgical treatment of spinal metastatic tumors aims to relieve pain, improve neurological symptoms, maintain spinal stability, and improve patients’ quality of life^2^. With the improvement of vertebroplasty, subtotal corpectomy, and total spinal resection, the scope of surgical resection and the relative survival benefit of patients are increasing. However, how to balance the trauma caused by surgery and maintain local control has become the focus of current research^3^. Therefore, the concept of "separation surgery" came into being, and the separation surgery was used as a surgical method to separate the anterior sulcus in the spinal canal from the posterior edge of the vertebral body^4^. Not only the dural compression can be relieved, but also the cerebrospinal fluid space around the spinal cord can be restored to build the radiotherapy gradient zone, and stereotactic body radiotherapy (SBRT) can be safely performed for severe ESCC cases caused by radiotherapy‐insensitive tumors^5^. As a radiotherapy method that can form a dose drop zone between the target zone and normal tissue, SBRT uses the principle of focusing, and with its features of precise stereotactic and multi‐path illumination, it has a huge technical advantage over traditional radiotherapy^6^. This feature is of great significance for the treatment of metastatic tumors in the spine, because it can maximize the dose of vertebral tumor radiation at the safe dose for the spinal cord. The upper limit of the safe dose of radiotherapy for the spinal cord is 14 Gy^7^, calculated according to the 50% decreasing dose gradient of the target area and the periphery^8^, which means that the theoretical maximum single dose of the vertebral body is 28 Gy. This dose can achieve ideal control of tumor under precise spatial localization. Therefore, after the appearance of SBRT, conventional radiotherapy can be performed for radiotherapy‐sensitive tumors, and the effect of traditional radiotherapy on spinal cord tissue can be avoided for radiotherapy‐insensitive tumors. The radiotherapy target area was designed according to the scope of tumor invasion of different patients with preoperative magnetic resonance imaging (MRI), and the dura border could be determined according to the computed tomography (CT) scan results of postoperative vertebral angiography, so that all kinds of spinal metastases can be satisfactorily controlled by SBRT^9^.

Therefore, this study collects follow‐up data including basic case, epidemiological characteristics, primary site, surgical procedure, symptom relief, pain assessment, and survival prognosis. It uses integrated analysis to study the effects of separation surgery and SBRT on prognosis and outcome in patients with spinal metastatic tumors in order to: (i) analyze whether patients with spinal metastatic tumors are suitable for separation surgery; (ii) implement the precautions for choosing the separation surgery; and (iii) summarize the related effects of separation surgery and SBRT.

## Materials and Methods

### *Clinical Data Collected by Single‐Center Treatment*

The cases of spinal metastatic tumors from January 2015 to December 2018 were collected according to the following inclusion criteria: (i) all cases with complete clinical data and follow‐up records were corrected by CT, MRI, and electroconvulsive therapy (ECT) to confirm the bone metastasis before surgery, imaging scan showed ESCC; (ii) all patients with spinal metastatic tumors were treated with separation surgery by routine, and part of patients accepted SBRT radiotherapy regimens of single high dose; (iii) Frankel neurological function grading, Karnofsky performance scores, VAS scores, ESCC grading and muscle strength grading were used to assess the patients’ condition; (iv) the scores of patients who accept surgery or SBRT and muscle strength grading were measured by t‐test. The Fisherʼs test was used among the multiple groups. The exclusion criteria were as follows: (i) SINS score <7 points (Table 1); (ii) ECOG score >3 points, cannot tolerate related surgical treatment; and (iii) the primary lesions were uncontrollable, and the estimated survival time of the patient was <3 months.

**Table 1:** Preoperative spinal stability score (SINS) of all the patients

Items	SINS	*n* (%)
**Location**		
Junctional (occiput‐C_2_, C_7_–T_2_, T_11_–L_1_, L_5_–S_1_)	3	15 (28.8)
Mobile spine (C_3_–C_6_, L_2_–L_4_)	2	17 (32.7)
Semirigid (T_3_–T_10_)	1	14 (27.0)
Rigid (S_2_–S_5_)	0	6 (11.5)
**Pain**		
Yes	3	46 (88.5)
Occasional pain but not mechanical	1	6 (11.5)
Pain‐free lesion	0	0 (0)
**Bone lesion**		
Lytic	2	21 (40.4)
Mixed (lytic/blastic)	1	29 (55.8)
Blastic	0	2 (3.8)
**Radiographic spinal alignment**		
Subluxation/translation present	4	17 (32.7)
Kyphosis/scoliosis	2	22 (42.3)
Normal alignment	0	13 (25)
**Vertebral body collapse**		
>50% collapse	3	14 (27)
<50% collapse	2	16 (30.7)
No collapse, >50% vertebra involved	1	20 (38.5)
None of the above	0	2 (3.8)
**Posterolateral involvement of spinal elements**		
Bilateral	3	20 (38.5)
Unilateral	1	31 (59.6)
None of the above	0	1 (1.9)

According to the criteria, SINS scores of 52 included patients were ≥ 7 points.

To develop a summary of the diagnosis and treatment of patients with spinal metastatic tumors, information was collected at a patient's first consultation, including gender, age, primary tumor type, tumor metastasis location, treatment (single separation surgery or separation surgery combined with precision radiotherapy), condition before and after treatment, recurrence and metastasis, no tumor time (counted in months), total survival time (counted in months), survival (death time) for data collection and classification statistics. Fifty two patients with spinal metastatic tumors treated with separation surgery were screened, including 24 males and 28 females, aged 25–77 years, with an average of 56.7 ± 7.4 years. Distribution of primary tumor type: 19 cases of lung cancer, eight cases of breast cancer, five cases of renal cancer, four cases of thyroid cancer, three cases of liver cancer, two cases of prostate cancer, one case of rectal cancer, one case of gastric cancer, one case of nasopharyngeal carcinoma, one case of foot rhabdomyosarcoma, and another seven cases of unknown origin. Distribution of tumor metastasis location: two cases of thoracic vertebra simply, 11 cases of lumbar vertebra simply, eight cases of sacral vertebra simply, and 31 cases of multiple bone metastasis (59.6%), including 15 cases of thoracic vertebrae with multiple metastases, 14 cases of lumbar vertebrae with multiple metastases, and atlas with multiple metastases in two cases. There were totally 17 cases of thoracic vertebrae, 25 cases of lumbar vertebrae, and 10 cases of atlas, including six cases of single‐segment in thoracic vertebrae, 18 cases of single‐segment in lumbar vertebrae, five cases of single‐segment in atlas, and 11 cases of multi‐segment in thoracic vertebrae, seven cases of multi‐segment in lumbar vertebrae, and five cases of multi‐segment in sacrum.

All the 52 patients had pain symptoms before surgery, and there were 28 patients with abnormal muscle strength (53.8%) before surgery, including three cases of grade 0, six cases of grade 1, two cases of grade 2, six cases of grade 3, and 11 cases of grade 4. Preoperative Frankel neurological function grading: four cases of grade A, 18 cases of grade B, 24 cases of grade C, six cases of grade D, zero cases of grade E. ESCC grading: one case of grade 1a, three cases of grade 1b, six cases of grade 1c, 24 cases of grade 2, and 18 cases of grade 3. The average VAS score was 7.28±1.91. Karnofsky performance scores: zero cases of 80–100 points, four cases of 50–70 points, and 48 cases of less than 50 points. The average score of Tomita was 5.7 ± 1.4 points, and the average score of Tokuhashi was 10.2 ± 2.8 points (Table 2).

**Table 2:** Preoperative scores and grading of all the patients

Scores and grading	Classification	Number (patients)
Frankel neurological function grading	grade A	2
	grade B	18
	grade C	26
	grade D	6
	grade E	0
Karnofsky performance scores	80–100 points	0
	50–70 points	4
	less than 50 points	48
Tomita scores	2–3 points	15
	4–5 points	26
	6–7 points	7
	8–10 points	4
Tokuhashi scores	0–8 points	6
	9–11 points	28
	≥12 points	18
VAS scores	0 point	0
	1–3 points	6
	4–6 points	28
	7–10 points	18
Epidural spinal cord compression grading (ESCC)	grade 0	0
	grade 1a	1
	grade 1b	3
	grade 1c	6
	grade 2	24
	grade 3	18
Muscle strength grading	grade 0	3
	grade 1	6
	grade 2	2
	grade 3	6
	grade 4	11
	grade 5	24

Frankel neurological function grading, ESCC grading, Muscle strength grading, Karnofsky performance scores, VAS scores, Tomita scores and Tokuhashi scores were used to assess the patientsʼ condition before treatment.

### *Surgical Methods*

All patients with spinal metastatic tumors underwent separation surgery. The approach was similar to the classic posterior pedicle approach, including: (i) General anesthesia, prone position. (ii) Revealing the spinal cord compression segment and the upper and lower adjacent lamina, the compression segment lamina is fully resected and decompressed, and the lateral block screw or pedicle screw posterior fixation is performed in at least two adjacent segments. After the posterior decompression, the articular process is removed through one or both sides of the pedicle approach, and the front of the dura is exposed. In order to reduce the decompression of the dura mater, the posterior longitudinal ligament should be removed together (ligament excision is generally performed in non‐tumor segments to better reveal the isolated dura mater). (iii) Carefully peel off the tumor tissue that is stuck in front of the dura mater. The frontal vertebral body destroyed by the tumor should be scraped off the tumor as much as possible, and the soft tissue should be removed such as the intervertebral disc, so as to achieve totally decompression around the compressed dura mater. (iv) If the vertebral body is affected by more than 50%, after removing the part of the vertebral body, a suitable titanium mesh should be placed in the anterior intervertebral and complete bone graft reconstruction. (v) Due to the presence of internal fixation, MRI examination will have artifacts, which will make it impossible to judge the precise position and boundary of the dura mater. Therefore, CT scan of the vertebral canal is routinely required after the separation surgery to determine the dura border.

### *SBRT*

SBRT is usually performed 10–20 days after surgery, and the target area design is generally 2–3 mm outside the tumor involvement range. According to the history of previous radiotherapy, radiotherapy sensitivity of tumor, ESCC classification, the number of paravertebral infiltration and affected segments, two different SBRT radiotherapy regimens can be selected: single high dose (24Gy) or high fractional dose (18–36Gy/3–6 times). Single high‐dose radiotherapy is suitable for most patients, and the upper dose limit is limited by the maximum radiation dose of the surrounding normal structure.

### *Each Parameter in Outcome Measures*

1. Frankel neurological function grading is divided into five grades. Grade A is the total loss of sensory and motor function. Grade B is the ability to retain only part of the sensory function and loss of exercise capacity. Grade C is the movement that must rely on external force to help but retain part of the movement. Grade D is normal walking but there is nerve damage. Grade E is normal function.
2. Karnofsky performance scores evaluate a patient's ability to survive for cancer. The evaluation of 80–100 points is the ideal functional status that can work independently and carry out daily life without special needs. An evaluation of 50–70 points is moderate, where a patient can provide their own self‐care, but cannot work, and some aspects of assistance are needed. An evaluation below 50 points is a poor functional state, where the patient cannot take care of themselves and requires a lot of medical help.
3. Tomita scores is a classic scoring system based on the patient's tumor metastasis model to predict the life of the patient and develop a targeted treatment plan. The total score is 10 points. The higher the score, the shorter the expected survival time. Total score of 2–3 points: long survival period (less than 2 years), good prognosis, long‐term local control, feasible wide or marginal resection. Total score of 4–5 points: mid‐life (between 1–2 years), mid‐term local control, feasible marginal resection or intralesional resection. Total score of 6–7 points: short survival period (between June and December), only short‐term local control, suitable for palliative surgery. Total score of 8–10 points: the end stage (less than 3 months), the prognosis is poor, suitable for non‐surgical treatment.
4. Tokuhashi scores add the weight of the patient's neurological function to the scoring model based on the Tomita score. The total score is 15 points. The higher the score, the longer the expected survival time. 0–8 points: survival period is expected to be ≤6 months, and conservative treatment is recommended. 9–11 points: survival is expected to be ≥6 months, and palliative surgery (including single lesions, no metastasis to the main internal organs) is recommended. ≥12 points: estimated survival period ≥12 months, recommended resection.
5. ESCC grading^11^ is divided according to the degree of tumor compression of the dura mater and spinal cord. Grade 0 is only destroying the bone and does not cause compression on the dura mater; Grade 1a is in contact with the dura mater but the dura mater is not deformed; Grade 1b is a dura mater that has been deformed and does not involve the spinal cord; Grade 1c is pressure contact with the spinal cord but no obvious compression of the spinal cord; Grade 2 is spinal cord compression but cerebrospinal fluid still surrounds the spinal cord; Grade 3 is that the spinal cord is pressed to one side and there is no cerebrospinal fluid around it.
6. VAS score is a rating scale that judges the degree of pain by the patient's intuitive experience. It set from 0 to 10 points, and 10 points is the most painful. 0 points: painless; 3 points or less: mild pain, tolerable; 4–6 points: the pain affects sleep, still tolerable; 7–10 points: strong pain, intolerable, affecting appetite and sleep.
7. Spinal Instability Neoplastic Score (SINS)^10^ is a scale based on tumor location, local pain, bone lesion properties, spinal force line, vertebral collapse, and posterolateral involvement of the spine, which assesses the degree of spinal instability and determines the timing of surgery^11^. The total score is 18 points. The higher the score, the more unstable the spine and the more the spinal balance should be reconstructed. According to the SINS score, 0–6 points: the spine is stable; 7–12 points: the spine is potentially unstable; 13–18 points: the spine is unstable; if the score exceeds 7 points, the surgery is necessary.

### *Statistical Processing*

All data were statistically analyzed by SPSS 23.0 (SPSS Inc., Chicago, IL, USA). The scores of patients who accept surgery or SBRT and muscle strength grading were measured by *t*‐test. The Fisherʼs test was used among the multiple groups. *P* < 0.01 was considered statistically significant. Kaplan‐Meier analysis and the Log⁃rank test were used to calculate the hazard ratio (HR) and the 95% feasible interval for patients with different ages, genders, and treatments. The multivariate Cox regression model was used to calculate the risk value HR and the 95% feasible interval (constrained age and gender adjusted for confounding factors) in patients undergoing only separation surgery or separation surgery combined with SBRT. The test level α value is 0.05 on both sides.

## Results

### *Changes in Postoperative Scores and Grading*

Fifty‐two patients were followed up for 9–47 months (mean 26.3±18.1 months, by telephone), including: (i) Frankel neurological function grading, Karnofsky performance scores, VAS scores, ESCC grading and muscle strength grading; (ii) recurrence and metastasis; and (iii) survival or death. Postoperative reexamination of CT and MRI had been done to observe local recurrence (1 month, 3 months, 6 months, and 1 year after surgery; every 6 months from the first year after surgery), and according to the pain outside the surgical area, all the patients were examined by imaging to identify new distant metastases. Separation surgery of all patients was successfully completed and followed up, and 15 patients died due to the deterioration of the primary tumor, including seven cases of lung cancer, one case of liver cancer, one case of breast cancer, and one case of foot rhabdomyosarcoma, and another five cases of unknown origin. The average time of operation was 158.3±155.9 min, and the average amount of intraoperative blood loss was 450.0 ± 80.8 mL. Forty‐six patients had pain relief (88.5%), six patients had no significant pain relief (11.5%), and postoperative muscle strength decreased in seven cases, muscle strength did not change in two cases, and muscle strength increased in 19 cases. Postoperative Frankel neurological function grading: one case of grade A, three cases of grade B, 22 cases of grade C, 21 cases of grade D, and five cases of grade E; Postoperative ESCC grading: 38 cases of grade 0, eight cases of grade 1a, two cases of grade 1b, three cases of grade 1c, and one case of grade 2 (Table 3). The average VAS score decreased to 2.17 ± 0.52 points, which was significantly improved compared with preoperative (*P* < 0.01). Karnofsky performance scores: six cases of 80–100 points, 38 cases of 50–70 points, and eight cases of less than 50 points, which was also significantly improved compared with preoperative scores (*P* < 0.01) (Table 4). There were three cases of postoperative complications, including two cases of wound infection which improved after anti‐infection and local debridement, and one case of cerebrospinal fluid leakage which improved after intraoperative repair of sutured dura mater and postoperative conservative treatment for 2 weeks.

**Table 3:** Postoperative scores and grading of all the patients

Scores and grading	Classification	Number (patients)
Frankel neurological function grading	grade A	1
	grade B	3
	grade C	22
	grade D	21
	grade E	5
Karnofsky performance scores	80–100 points	6
	50–70 points	38
	less than 50 points	8
VAS scores	0 point	6
	1–3 points	36
	4–6 points	4
	7–10 points	6
Epidural spinal cord compression grading (ESCC)	grade 0	38
	grade 1a	8
	grade 1b	2
	grade 1c	3
	grade 2	1
	grade 3	0
Muscle strength grading	grade 0	1
	grade 1	3
	grade 2	2
	grade 3	2
	grade 4	4
	grade 5	40

According to the clinical improvement (the improvement of syndromes and gradings) and functional evaluation (scores), Frankel neurological function grading, ESCC grading, Muscle strength grading, Karnofsky performance scores and VAS scores were used to assess the patientsʼ condition after surgery.

**Table 4:** Comparison of preoperative and postoperative scores of all patients

Scores	Preoperation	Postoperation	*P* value
VAS scores	7.28 ± 1.91	2.17 ± 0.52	0.0047
Karnofsky performance scores	30.25 ± 17.35	62.15 ± 14.75	0.0061

The 52 patientsʼ postoperative Karnofsky performance scores and VAS scores were significantly improved compared with preoperative scores (*P*<0.01).

### *Changes in Postoperative Scores and Grading of Patients with Multiple Bone Metastasis*

There were 31 cases of multiple bone metastasis before surgery (59.6%). The patients were followed up for 11–42 months (24.1 ± 13.2 months), and 11 patients died due to the deterioration of the primary tumor (11/31, 35.5%), including seven cases of lung cancer and one case of foot rhabdomyosarcoma, and another three cases of unknown origin. Twenty‐five patients had pain relief (80.6%), but six patients had no pain relief (19.4%). There were 21 patients with abnormal muscle strength (67.7%) before surgery, including three cases of grade 0, one case of grade 1, one case of grade 2, six cases of grade 3, and 10 cases of grade 4, and postoperative muscle strength decreased in three cases, muscle strength did not change in five cases, and muscle strength increased in 13 cases. Preoperative Frankel neurological function grading: four cases of grade A, 12 cases of grade B, 14 cases of grade C, one cases of grade D, zero cases of grade E. The average VAS score was 7.16 ± 1.85 before surgery. Karnofsky performance scores: zero cases of 80–100 points, two cases of 50–70 points, and 29 cases of less than 50 points. After surgery, postoperative Frankel neurological function grading: one case of grade A, two cases of grade B, 11 cases of grade C, 14 cases of grade D, three cases of grade E. The average VAS score decreased to 2.03 ± 0.34, which was significantly improved compared with preoperative (*P* < 0.01). Karnofsky performance scores: three cases of 80–100 points, 25 cases of 50–70 points, and three cases of less than 50 points, which was also significantly improved compared with preoperative (*P* < 0.01) (Table 5).

**Table 5:** Comparison of preoperative and postoperative scores of patients with multiple bone metastasis

Scores	Preoperation	Postoperation	*P* value
VAS scores	7.16 ± 1.85	2.03 ± 0.34	0.0059
Karnofsky performance scores	28.05 ± 10.25	68.31 ± 19.44	0.0036

The postoperative Karnofsky performance scores and VAS scores of 31 patients with multiple bone metastasis were significantly improved compared with preoperative scores (P<0.01).

### *Changes in Postoperative Scores and Grading of Patients with Unknown Origin*

There were seven cases of unknown origin (13.5%). These seven patients were followed up for 12–40 months (25.3 ± 11.7 months), and five patients died due to the deterioration of general condition and multiple organ failure (5/7, 71.4%). All the seven patients had pain relief (100%). There were three patients with abnormal muscle strength (42.9%) before surgery, including one case of grade 3, and two cases of grade 4, and postoperative muscle strength increased in all three cases. Preoperative Frankel neurological function grading: two cases of grade A, two cases of grade B, three cases of grade C, zero cases of grade D, zero cases of grade E. The average VAS score was 7.91 ± 1.56 before surgery. Karnofsky performance scores: zero cases of 80–100 points, one case of 50–70 points, and six cases of less than 50 points. After surgery, postoperative Frankel neurological function grading: zero cases of grade A, zero cases of grade B, four cases of grade C, two cases of grade D, one case of grade E. The average VAS score decreased to 1.62 ± 0.28, which was significantly improved compared with preoperative (*P* < 0.01). Karnofsky performance scores: two cases of 80–100 points, five cases of 50–70 points, and zero cases of less than 50 points, which was also significantly improved compared with preoperative (*P* < 0.01) (Table 6). However, the average time of operation was 202.4 ± 41.6 min, which was significantly longer than that of the patients with clear origin (*P* < 0.01). and the average amount of intraoperative blood loss was 613.5 ± 101.5 ml, which was significantly increased compared to that of the patients with clear origin (*P* < 0.01) (Table 7).

**Table 6:** Comparison of preoperative and postoperative scores of patients with unknown origin

Scores	Preoperation	Postoperation	*P* value
VAS scores	7.91 ± 1.56	1.62 ± 0.28	0.0058
Karnofsky performance scores	27.82 ± 14.77	60.08 ± 11.37	0.0019

The postoperative Karnofsky performance scores and VAS scores of 31 patients with unknown origin were significantly improved compared with preoperative scores (*P* < 0.01).

**Table 7:** Comparison of surgical time and intraoperative blood loss in all patients

Indexes	Cases of clear origin	Cases of unknown origin	*P* value
Surgical time (min)	136.5 ± 41.6	202.4 ± 41.6	0.0023
Intraoperative blood loss (mL)	388.5 ± 76.5	613.5 ± 101.5	0.0012

Surgical time and the intraoperative blood loss of patients with unknown origin were significantly increased than that of patients with clear origin (*P*<0.01).

### *Changes in Scores and Grading of Patients that Accepted Surgery Combined with SBRT*

There were 13 patients that accepted surgery combined with SBRT. The patients were followed up for 11–38 months (22.5 ± 10.2 months, by telephone), including: (i) Frankel neurological function grading, Karnofsky performance scores, VAS scores, ESCC grading and muscle strength grading; (ii) recurrence and metastasis; and (iii) survival or death. SBRT of all patients was successfully completed and followed up, and five cases (5/13) died of primary tumors, including three cases of lung cancer, one case of breast cancer, and one case of foot rhabdomyosarcoma. Twelve patients had pain relief (12/13), and the muscle strength decreased in zero cases, the muscle strength did not change in five cases, and the muscle strength increased in eight cases. After surgery and SBRT, Frankel neurological function grading: zero cases of grade A, zero cases of grade B, three cases of grade C, four cases of grade D, six cases of grade E; ESCC grading: 10 cases of grade 0, one case of grade 1a, two cases of grade 1b, zero cases of grade 1c, zero cases of grade 2, and zero cases of grade 3. The average VAS score decreased to 1.64 ± 0.41, which was significantly improved compared with preoperative and postoperative (*P* < 0.01). Karnofsky performance scores: eight cases of 80–100 points, four cases of 50–70 points, and one case of less than 50 points (Table 8), which was also significantly improved compared with preoperative and postoperative (*P* < 0.01) (Table 9).

**Table 8:** Scores and grading of patients accepted surgery & SBRT

Scores and grading	Classification	Number (patients)
Frankel neurological function grading	grade A	0
	grade B	0
	grade C	3
	grade D	4
	grade E	6
Karnofsky performance scores	80–100 points	8
	50–70 points	4
	less than 50 points	1
VAS scores	0 point	3
	1–3 points	7
	4–6 points	2
	7–10 points	1
ESCC grading (ESCC)	grade 0	10
	grade 1a	1
	grade 1b	2
	grade 1c	0
	grade 2	0
	grade 3	0
Muscle strength grading	grade 0	0
	grade 1	0
	grade 2	0
	grade 3	2
	grade 4	2
	grade 5	9

According to the clinical improvement (the improvement of syndromes and gradings) and functional evaluation (scores), Frankel neurological function grading, ESCC grading, Muscle strength grading, Karnofsky performance scores and VAS scores were used to assess the patientsʼ condition after surgery & SBRT.

**Table 9:** Comparison of scores of all patients before surgery, after surgery, and after surgery & SBRT

Scores	Preoperation	Postoperation	After operation+SBRT	*P* value
VAS scores	7.28 ± 1.91	2.17 ± 0.52	1.64 ± 0.41	0.0031 0.0020
Karnofsky performance scores	30.25 ± 17.35	62.15 ± 14.75	70.05 ± 18.25	0.0048 0.0062

The Karnofsky performance scores and VAS scores of 13 patients who accepted surgery combined with SBRT were significantly improved compared with preoperative and postoperative (*P* < 0.01).

### *Differences Between the Patients that Accepted Simple Separation Surgery and Separation Surgery Combined with SBRT*

By the end of the follow‐up, 13 patients had local recurrence after resection, and the recurrence rate was 25% (13/52). There were 10 cases of new distant metastasis, and the metastasis rate was 19.2% (10/52). After simple separation surgery (39 cases) and separation surgery combined with SBRT (13 cases), local recurrence was 12 cases and one case respectively, and the recurrence rate was 30.8% (12/39) and 7.7% (1/13). The new distant metastasis was 10 cases and zero cases respectively, and the rate of distant metastasis was 25.6% (10/39) and 0% (0/13). The average time of recurrence after simple separation surgery was 4.1 ± 1.2 months, and the average time of new metastasis was 7.2 ± 2.5 months. Only one patient after separation surgery combined with SBRT showed local recurrence 8 months after treatment. (Table 10) However, due to the small sample size, there had been inevitable errors in calculating and contrasting recurrence rates and metastasis rates of patients who accepted simple separation surgery and separation surgery combined with SBRT.

**Table 10:** Comparison of recurrence and metastasis after only separation surgery and separation surgery combined with SBRT

Indexes	Only separation surgery	Separation surgery + SBRT
Total number	39	13
Local recurrence number	12	1
New metastasis number	10	0
Recurrence rate (%)	30.8	7.7
Metastasis rate (%)	25.6	0
Average time of recurrence (months)	4.1 ± 1.2	8
Average time of metastasis (months)	7.2 ± 2.5	0
Death number	10	5

The recurrence and metastasis number of patients who accepted only separation surgery were more than the number of patients who accepted separation surgery combined with SBRT.

### *Univariate and Multivariate Analysis of Factors Affecting Overall Survival*

Kaplan‐Meier analysis and Log⁃rank test showed that the median survival time was 38 months for patients undergoing separation surgery combined with SBRT, and 21 months for patients undergoing simple separation surgery, and the difference was statistically significant (*P* = 0.002). There was no significant correlation between age, gender, metastasis, primary tumor type, and overall survival (OS) in this group (Table 11). Multivariate Cox proportional hazards regression analysis showed that separation surgery combined with SBRT was an independent predictor of OS (*P* = 0.001) (Table 12).

**Table 11:** Associations between selected variables and survival time of patients

Variables	Number (patients)	Number (deaths)	Overall survival (OS)		
			Median OS (months)	Log‐rank *P* *	*HR* (95% *CI*)†
Age (years)					
≤59	30	6 (20.0%)	25		1
>59	22	9 (40.9%)	23	0.734	0.94 (0.53–1.62)
Sex					
Male	24	7 (29.2%)	27		1
Female	28	8 (28.6%)	26	0.603	1.02 (0.60–1.81)
Metastasis					
Multiple	31	11 (35.5%)	24		1
Single	21	4 (19.0%)	23	0.741	0.84 (0.46–1.59)
Primary tumor					
Unknown origin	7	5 (71.4%)	20		1
Clear origin	45	10 (22.2%)	22	0.625	1.05 (0.54–1.76)
SBRT					
No	39	10 (25.6%)	21		1
Yes	13	5 (38.5%)	38	0.002	0.47 (0.32–0.82)

* Log‐rank *P* value was analyzed by Kaplan‐Meier analysis.

† *HR* was analyzed by Cox regression model.

**Table 12:** Multivariable Cox analysis for various diagnostic factors in patients with spinal metastatic tumor

Factors	Overall survival (OS)*		
	*HR*	95% *CI* of HR	*P* value
Primary tumor (Clear origin)	0.73	0.41–1.67	0.658
Metastasis (Single)	0.86	0.55–1.81	0.747
SBRT (Yes)	0.41	0.29–0.80	0.001

* Adjusted by age and sex in Cox regression analysis.

## Discussion

The clinical treatment of patients with spinal metastatic tumors needs to consider the surgical effect, the length of recovery, and the length of postoperative survival. Therefore, the systematic evaluation of the disease before the treatment being selected is crucial. Early Tomita scores^12^ and Tokuhashi scores^13^ are important for assessing the extent of tumor progression, surgical procedures, and predictive surgical outcomes. The preoperative average Tomita score of this group was 5.7 ± 1.4 points, and the preoperative average Tokuhashi score was 10.2 ± 2.8 points. All the patients were expected to have a longer survival period and a better prognosis, and were suitable for palliative and marginal resection or intralesional resection. However, these scores were developed under the traditional radiotherapy technology. With the emergence of the revolutionary technology of SBRT, the NOMS evaluation system has gradually become the latest evaluation and treatment decision‐making system for spinal metastatic tumors. Bilsky and his colleagues at the Memorial‐Sloan Kettering Cancer Center^14^ proposed this system as the only evaluation system for the treatment of spinal metastatic tumors under current radiotherapy technology. This evaluation system includes four aspects: neurologic, oncologic, mechanical instability, and systemic disease. Neurological function was assessed according to ESCC grading^15^; oncology characteristics were assessed based on sensitivity to radiotherapy; spinal stability was assessed according to the spinal in‐stability neoplastic score^16^; the general condition and degree of metastasis were assessed based on body tolerance to surgery and the Karnofsky performance scores. The surgeon also needs to consider the following factors when doing preoperative evaluation: (i) choose the suitable surgical methods for patients with spinal metastatic tumors; (ii) find the best radiotherapy regimens after surgery and whether the patient needs surgical intervention; and (iii) which type of surgical intervention is appropriate for the patient. Therefore, the surgical indications can be fully understood and strictly grasped in order to: (i) analyze whether patients with spinal metastatic tumors are suitable for separation surgery; (ii) implement the effect of separation surgery and SBRT; and (iii) summarize the relationship between SBRT and separation surgery.

### *Surgical Methods for Spinal Metastatic Tumors*

At present, surgical methods for spinal metastatic tumors include: partial appendix of vertebra resection, effective spinal canal decompression, direct injection of bone cement to the vertebral body through a pedicle, and fixation by the posterior nail system. Especially for elderly patients with high risk of surgery, Gerszten *et al*.^17^ used vertebral body formation combined with cyber knife to treat spinal metastases with vertebral height loss and angular deformity^18^. The pain relief rate was 92%. Therefore, our study rigorously screened patients with poor spine stability, good tolerance to surgery and SBRT by SINS score, ECOG score, metastasis, and estimated survival time. Compared with conditions before treatment, patients can obviously benefit from this procedure, as shown by: (i) the patient's subjective symptoms and VAS scores indicate that the pain is significantly relieved compared with preoperative; (ii) ESCC grading, Frankel neurological function grading, limb muscle strength after the dural compression is significantly improved compared with preoperative; (iii) Karnofsky performance scores indicate that the quality of life was significantly improved compared with preoperative; and (iv) by the end of the follow‐up, 13 patients had local recurrence after resection, and the recurrence rate was 25% (13/52). There were 10 cases of new distant metastasis, and the metastasis rate was 19.2% (10/52).

### *Precautions for Choosing Separation Surgery*

Patients with spinal metastatic tumors are mostly advanced tumors, and they are often involved in the multi‐segmental lesions of the spine. Therefore, our treatment no longer focuses solely on prolonging life, but more on the improvement in quality of patient life as the core principle of choosing a treatment plan. Treatment, therefore, requires the consideration of a variety of factors, including the patient's age, symptoms, tumor type, surgical tolerance, physical status and expected survival^19^. According to our results, there were 31 patients of multiple metastases before surgery – compared and observed from several aspects, such as subjective symptoms of pain, VAS score, ESCC grading after dural compression, Frankel neurological function grading, limb muscle strength, and Karnofsky performance scores – that significantly improved from their preoperative condition. This improvement was attributed to our strict screening of patients by SINS score, ECOG score, metastasis, estimated survival time and other factors before surgery; on the other hand, it was also attributed to "separation surgery" which could relieve symptoms, restore spinal stability and (the surgical concept of) minimize intraoperative trauma. According to the results of preoperative examination, separation surgery was performed on the segment that caused pain, severe ESCC and spinal instability with pathological fracture^20^. For spinal metastatic tumors with a wide range of lesions, as long as the key position was selected according to the indications, satisfactory surgical goals can still be achieved, even if this is only the improvement of the patient's quality of life.

This study also summarized the treatment of patients with spinal metastatic tumors of unknown primary origin. The results showed that those seven patients benefited from pain relief, improvement of neurological symptoms, and improvement of quality of life after treatment. However, the average time of operation was 202.4 ± 41.6 min, which was significantly longer than that of the patients with clear origin (*P* < 0.01) and the average amount of intraoperative blood loss was 613.5 ± 101.5 mL, which was significantly increased compared to that of the patients with clear origin (*P* < 0.01). The prolongation of operation time is related to insufficient preoperative preparation and intraoperative hemorrhage, and the use of preoperative selective arterial embolization intervention can effectively reduce intraoperative blood loss^21^. Five patients died due to the deterioration of general condition and multiple organ failure (5/7, 71.4%). It is further suggested that for patients with metastatic tumors of the primary lesion whose origin is unknown, the prognosis is poor, and sufficient preoperative preparation and evaluation should be carried out.

### *The Advantages of Separation Surgery Combined with SBRT for Spinal Metastatic Tumors*

Compared to traditional eternal beam radiation therapy (EBRT), SBRT and stereotactic radiosurgery (SBRT) can take advantage of advanced imaging systems, planning software, image‐guided positioning, and intensity‐adjusted dose delivery, accurately emitting high‐density ray energy to the target area. This can cause a sharp drop in dose between the target and adjacent normal tissue, not only increasing the radiation dose from a single shot to the target area, but also ensuring that the surrounding tissues (especially the spinal cord and the horsetail) are exposed to safe doses, which can better control the pain of patients with spinal metastatic tumors and the neurological symptoms caused by spinal cord compression. Our study found that 12 of the 13 patients who received SBRT had significant relief of pain symptoms, and their VAS pain scores were significantly improved when comparing preoperative and postoperative examinations (*P* < 0.01). In terms of relieving neurological symptoms caused by spinal cord compression and improving quality of life, ESCC grading, Frankel neurological function grading, limb muscle strength, and Karnofsky performance scores of patients who accepted separation surgery combined with SBRT were significantly improved when comparing preoperative and postoperative examinations. Kaloostian *et al*.^22^ performed a meta‐analysis of the efficacy of SBRT in 1028 patients and found that the pain relief rate was 83% and the local tumor control rate was 92%. Gerszten *et al*.^23^ collected 500 patients with spinal metastases who underwent SBRT treatment. Among patients who received SBRT for the first time, the local tumor control rate could reach 90%. Ryu *et al*.^24^ observed in a prospective study that the volume of tumors that were compressed on the dura at 2 months after SBRT treatment was reduced by an average of 65% and the neurological improvement rate was 81%. For patients who have failed to receive conventional radiotherapy, patients who have received SBRT again can achieve local tumor control rate of 80% within 1 year.

Moulding *et al*.^25^ first proposed posterior decompression surgery combined with single high‐dose SBRT in 21 patients with spinal metastases in 2010, and obtained a local tumor control rate of 81%. However, the concept of “separation surgery” wasn't officially proposed by Laufer *et al*. until 2013. All the 186 patients they collected underwent separation surgery combined with postoperative high fractional dose radiotherapy or single high‐dose radiotherapy, and achieved a local tumor control rate of 83.6% within 1 year. Mantel *et al*.^26^ found that radiotherapy dose affects local tumor progression by single factor analysis, and satisfactory local tumor control can be achieved regardless of the primary tumor type and SBRT radiotherapy regimens, which is consistent with our findings. A study by Katsoulakis *et al*.^27^ of the postoperative pathological results of 32 cases with spinal metastases showed that 78% of the lesions that accepted SBRT with a single dose of 18‐24 Gy could achieve a radical effect, with only a small number of lesions (22%) having pathological residues. Our study further found that the recurrence rate and metastasis rate after separation surgery combined with SBRT precision radiotherapy were significantly lower than those of simple separation surgery (*P* < 0.01), especially in the absence of new metastasis. At the same time, only one patient developed local recurrence 8 months after treatment, and the time to recurrence was significantly delayed compared with patients who only underwent separation surgery. Moreover, Our study showed that the median survival time of patients undergoing separation surgery combined with SBRT was significantly longer than patients undergoing simple separation surgery (*P* = 0.002). Multivariate Cox proportional hazards regression analysis also showed that separation surgery combined with SBRT (*P* = 0.001) was an independent predictor of OS. Ryu *et al*.^28^ demonstrated that a single high‐dose (16 Gy) SBRT was safe and effective in 44 patients with spinal metastases. According to the results of their study, the median pain relief time was 2 weeks, and the long‐term pain control rate was 80%–90%, and the recurrence rate of spinal metastases in the surrounding tissues is lower than 5%.

At the same time, the separation surgery combined with SBRT has a low incidence of post‐radiation complications due to the small amount of radiation to surrounding normal tissues. In our study, only three complications occurred after surgery, and they were all not associated with acute and chronic adverse reactions of spinal cord irradiation. A retrospective analysis by Hall *et al*.^29^ showed that only 0.5% of 1388 patients with spinal metastases treated with SBRT developed radioactive myelopathy. In addition, Stubblefield *et al*.^30^ showed that peripheral neurological damage occurred at a dose of 24 Gy for a single SBRT of the cervical and lumbar spine, but the overall incidence was only 2.5%. Heron *et al*.^31^ compared the results of a single SBRT and multiple SBRT using a jet knife, and found that there was no significant difference in the incidence of adverse reactions between the two different regimens.

In summary, surgical treatment of spinal metastatic tumors has received increasing attention in the industry^32^. The preoperatively designed separation surgery has a small risk of intraoperative trauma and postoperative complications, and can be combined with SBRT. It can not only significantly improve patients’ pain and nerve function, and relieve spinal cord compression, but can also significantly improve the quality of lives of the patients with end‐stage cancer, so as to obtain better local treatment effect and systemic improvement. For spinal metastatic tumors with wide range of lesions, it is necessary to select the key position to achieve the purpose of surgery; for patients with spinal metastatic tumors of unknown origin, preoperative scores and grading, interventional embolization, and evaluation of prognosis should be carried out. However, these conclusions still need to be confirmed by a larger sample size study.

**Figure 1:**
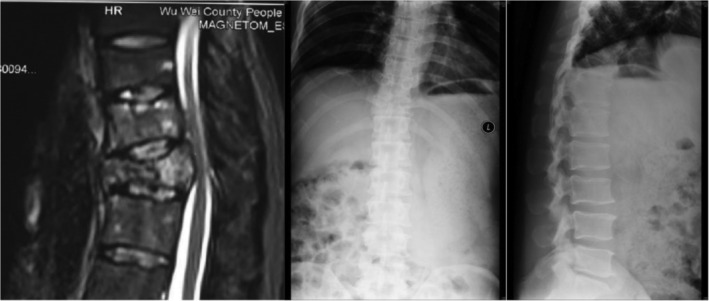
Preoperative MRI and X‐ray scan.

**Figure 2:**
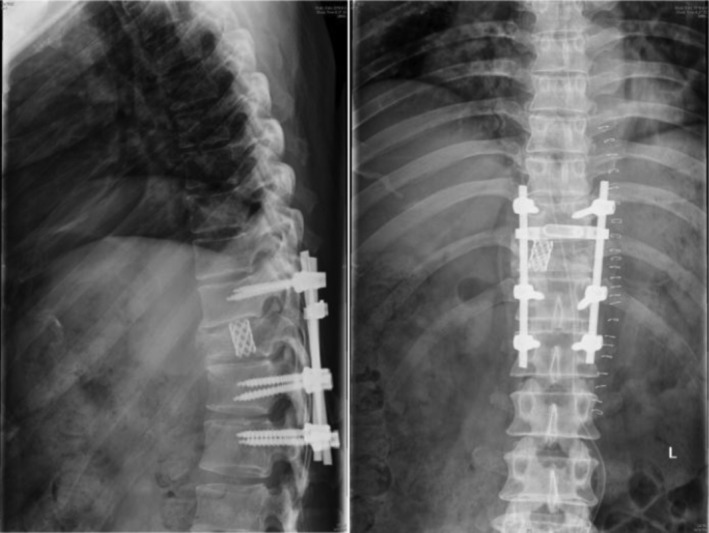
Postoperative X‐ray scan.

**Figure 3:**
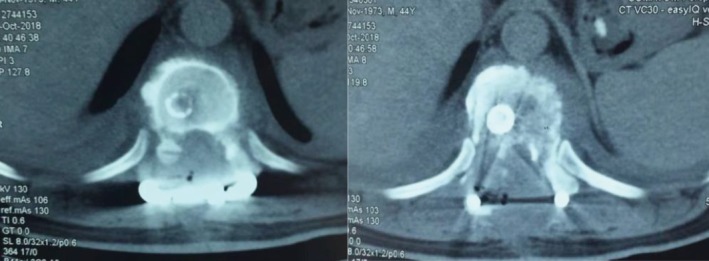
CT Scan before SBRT precision radiotherapy.

**Figure 4:**
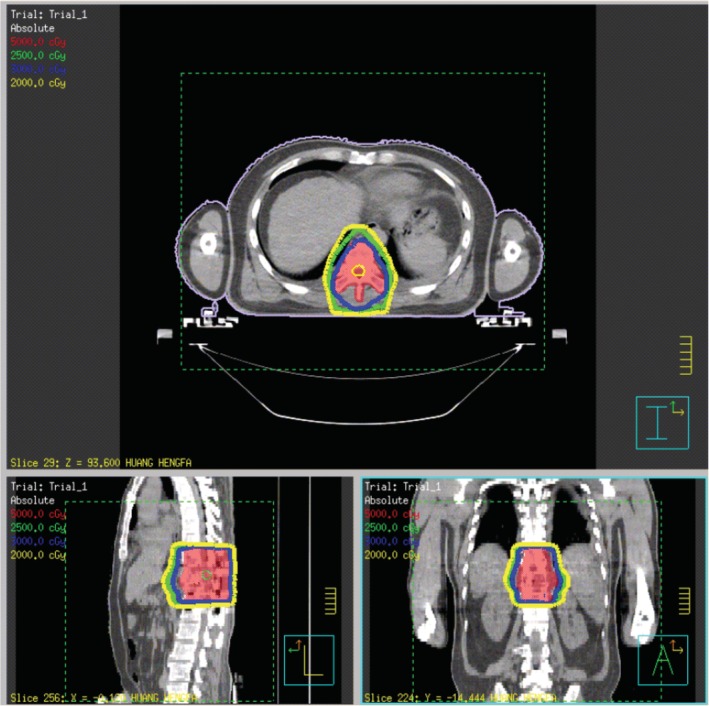
SBRT precision radiotherapy.

**Figure 5:**
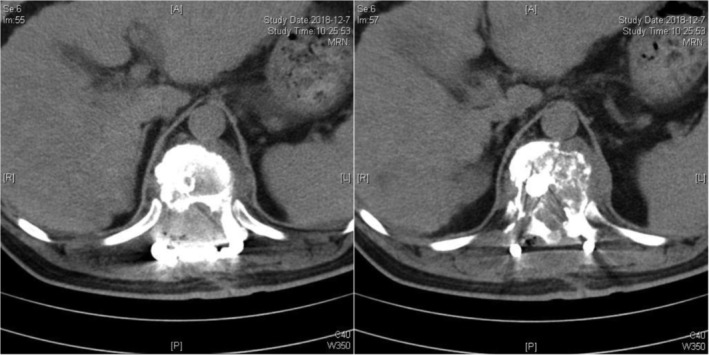
CT Scan after SBRT precision radiotherapy (2 months).

Case 1 Male, 45 years old, liver cancer with lung metastasis, multiple spine metastasis (T_11_ obvious), back pain with incomplete paralysis (Figs 1, 2, 3, 4, 5).

**Figure 6:**
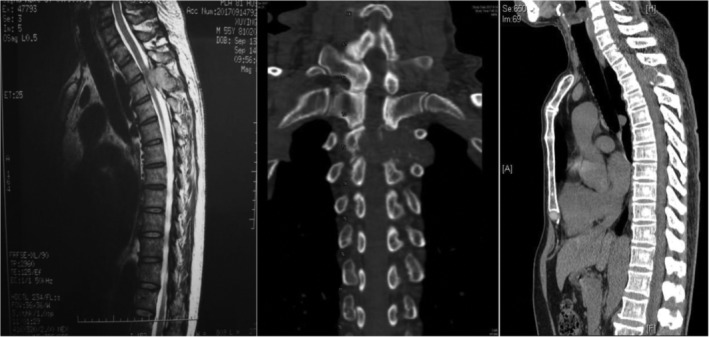
Preoperative MRI and CT scan.

**Figure 7:**
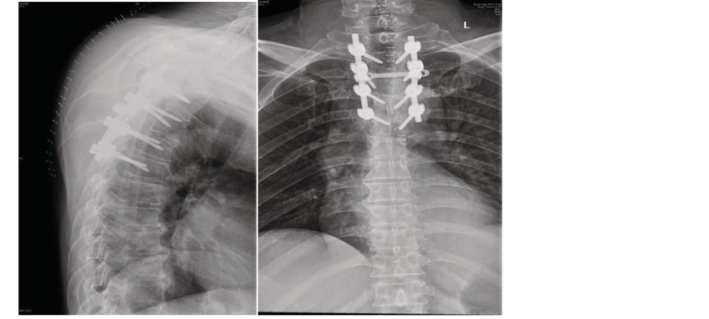
Postoperative X‐ray scan.

**Figure 8:**
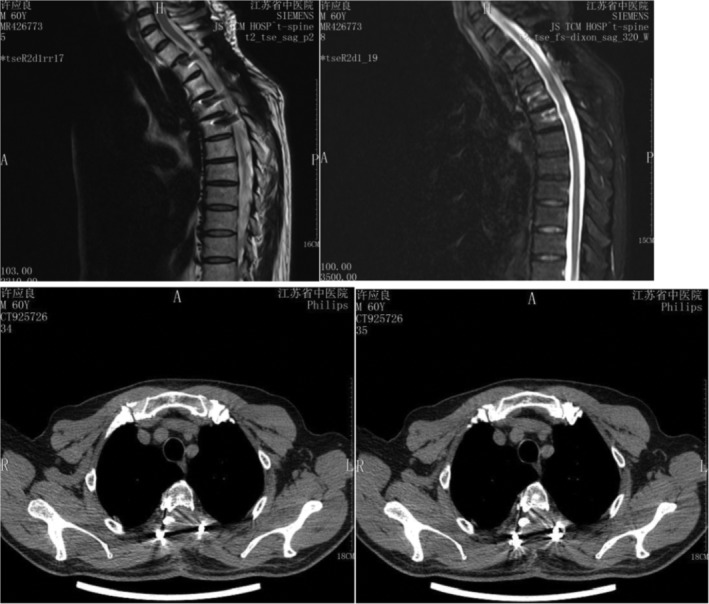
MRI and CT scan before SBRT precision radiotherapy.

**Figure 9:**
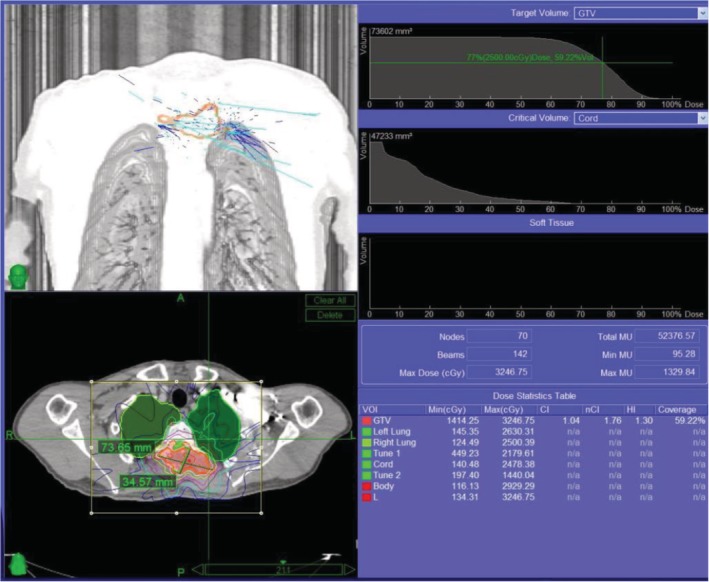
SBRT precision radiotherapy.

**Figure 10:**
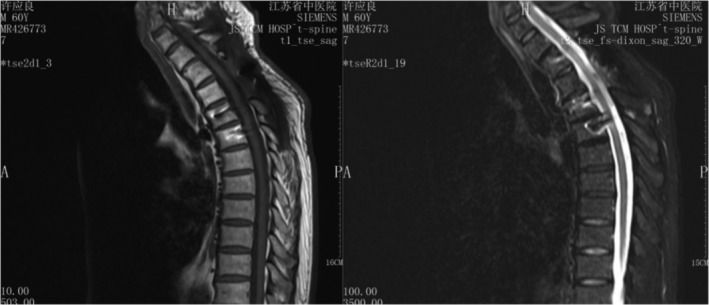
MRI and CT scan after SBRT precision radiotherapy (3 months).

Case 2 Male, 55 years old, thyroid cancer with simple spine metastasis (T_3_), back pain with incomplete paralysis (Figs 6, 7, 8, 9, 10).

**Figure 11:**
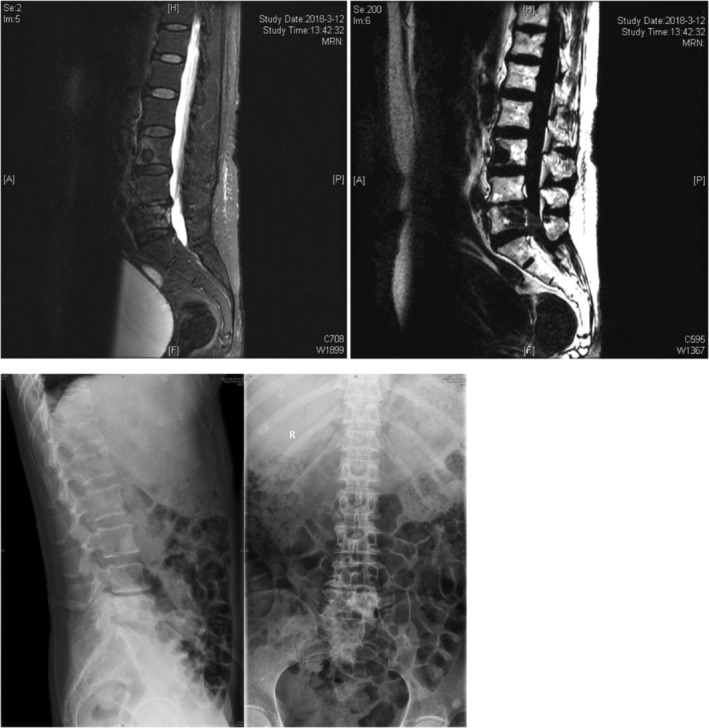
Preoperative MRI and X‐ray scan.

**Figure 12:**
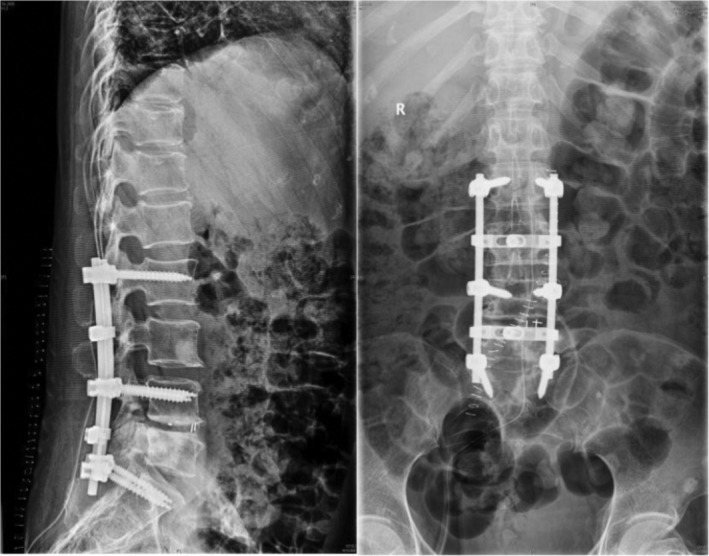
Postoperative X‐ray scan.

**Figure 13:**
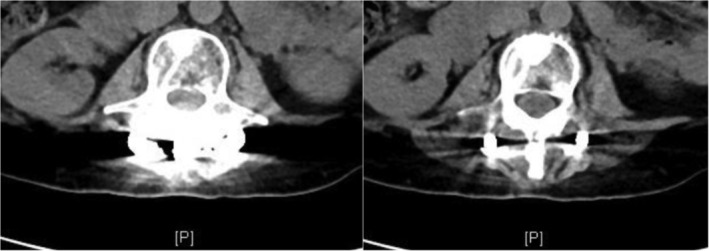
CT Scan before SBRT precision radiotherapy.

**Figure 14:**
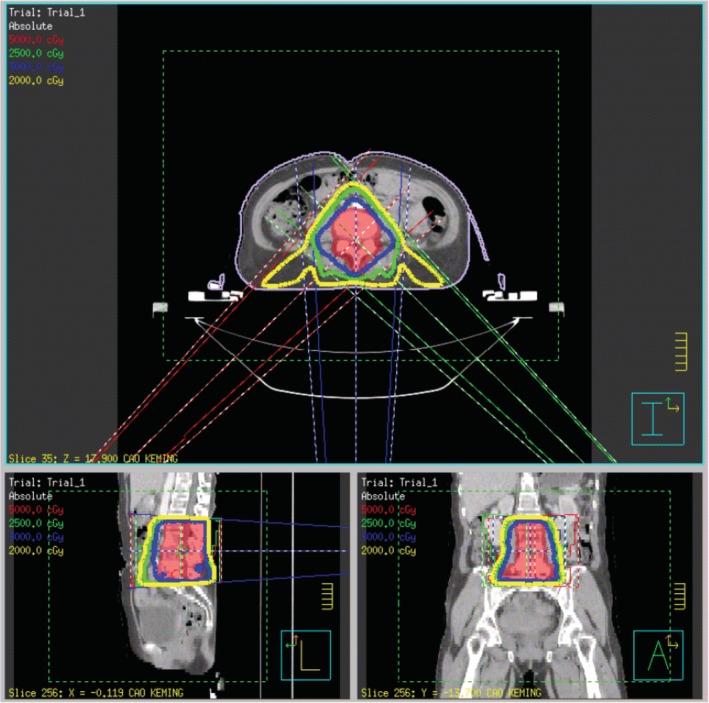
SBRT precision radiotherapy.

**Figure 15:**
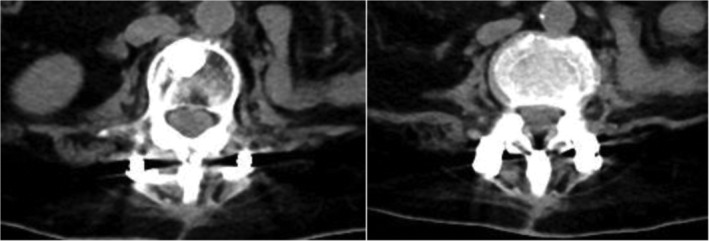
CT Scan after SBRT precision radiotherapy (2.5 months).

Case 3 Female, 58 years old, lung cancer with adrenal gland metastasis, multiple spine metastasis (L_3_, L_5_ obvious), lower back pain with complete paralysis (Figs 11, 12, 13, 14, 15).

After separation surgery, pain and spinal cord compression symptoms of the patients above were significantly relieved, various scores and limb muscle strength were significantly improved. The patient accepted SBRT precision radiotherapy within 2–3 months after surgery. We further found that it cannot only obtain better local treatment effect, but also significantly improve the quality of a patient's life.
